# Direct Cellularity Estimation on Breast Cancer Histopathology Images Using Transfer Learning

**DOI:** 10.1155/2019/3041250

**Published:** 2019-06-09

**Authors:** Ziang Pei, Shuangliang Cao, Lijun Lu, Wufan Chen

**Affiliations:** School of Biomedical Engineering and Guangdong Provincial Key Laboratory of Medical Image Processing, Southern Medical University, Guangzhou 510515, China

## Abstract

Residual cancer burden (RCB) has been proposed to measure the postneoadjuvant breast cancer response. In the workflow of RCB assessment, estimation of cancer cellularity is a critical task, which is conventionally achieved by manually reviewing the hematoxylin and eosin- (H&E-) stained microscopic slides of cancer sections. In this work, we develop an automatic and direct method to estimate cellularity from histopathological image patches using deep feature representation, tree boosting, and support vector machine (SVM), avoiding the segmentation and classification of nuclei. Using a training set of 2394 patches and a test set of 185 patches, the estimations by our method show strong correlation to those by the human pathologists in terms of intraclass correlation (ICC) (0.94 with 95% CI of (0.93, 0.96)), Kendall's tau (0.83 with 95% CI of (0.79, 0.86)), and the prediction probability (0.93 with 95% CI of (0.91, 0.94)), compared to two other methods (ICC of 0.74 with 95% CI of (0.70, 0.77) and 0.83 with 95% CI of (0.79, 0.86)). Our method improves the accuracy and does not rely on annotations of individual nucleus.

## 1. Introduction

Breast cancer is the most common malignant cancer occurring in women [1]. Preoperative neoadjuvant therapy (NAT) [2] can reduce the breast tumor size, so as to facilitate the complete resection of tumor and the performance of breast-conserving surgery instead of mastectomy for patients with large tumor. With NAT, a significant reduction of recurrence and metastasis can be achieved [3]. Pathologic complete response (pCR) [4] has been conventionally accepted as the primary end point to evaluate the efficacy of NAT [5]. However, the predictive potential of pCR on long-term prognosis is impaired by its blurry definition (e.g., there is no agreement on whether pCR should be also applied to the axillary lymph node and whether the presence of only noninvasive cancer should be defined as pCR) [4] and the roughness of dichotomizing the tumor response (as complete response or residual disease) [5].

Unlike pCR, the residual cancer burden (RCB) index is improved by measuring both in situ and invasive cancer in residual tumor and the metastasis through lymph nodes [5]. RCB is a new staging system basically devised to continuously quantify the residual breast cancer that ranges from complete response to chemotherapy resistance. It is standardized by defining a pipeline of specimen collection and tumor bed identification and has proved to be a significant indicator of distant relapse-free survival of breast cancer [5].

**Figure 1:**
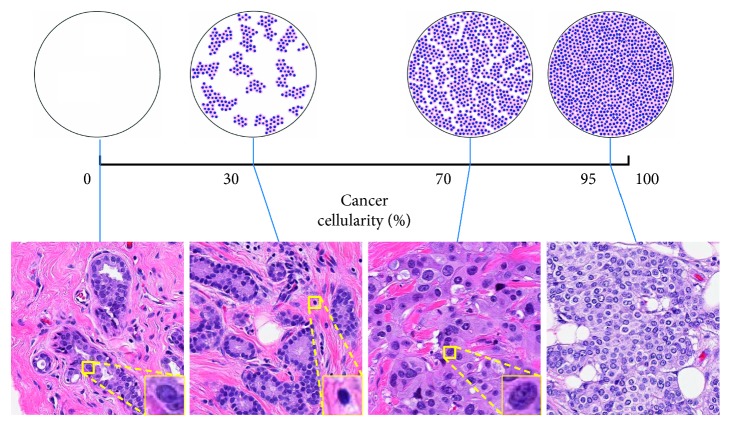
Illustrations of the definition of cellularity. In the top row are 4 computer generated diagrams [6] used as reference for manual estimation. In the bottom row are 4 example patches from the test set labeled with manually estimated cellularity and 3 zooming windows showing the morphology of benign epithelial, lymphocyte, and malignant epithelial nuclei.

Clinically, RCB is assessed by checking the histological sections from primary breast tumor site and regional lymph nodes [6]. The RCB index is calculated using six parameters, namely, the primary tumor bed area (length and width), the overall cancer cellularity, the percentage of in situ cancer, the number of positive lymph nodes, and the diameter of the largest metastasis [6]. Among them, the estimation of cancer cellularity is a critical and challenging task. Cancer cellularity is defined as the proportion of cancer within the residual tumor bed. In clinical practice, the largest cross-sectional area of the preidentified tumor bed is divided into multiple slides, which are stained with hematoxylin and eosin (H&E) and then reviewed with a microscope. In each microscopic field, a pathologist estimates the local cellularity by comparing the proportion of area containing cancer to the standard reference. The top row of Figure 1 presents some of the computer-generated diagrams that illustrate the distribution of cancerous nuclei under different cellularity and can be used as references to assist manual estimation. The overall cellularity is then obtained by averaging these manual estimations over all the fields. However, the reliability of manual assessment on tumor cell percentage is subject to inter-rater variability [7], and the procedure is time-consuming and also requires expertise and experience.

These problems can be probably solved using computerized methods. Digital pathology [8, 9] has enabled software to retrieve useful information from digitized slides that could be further analyzed using advanced statistical learning models. Combined with machine learning, digital pathology has been developed as a powerful tool in various clinical applications such as histological classification [10, 11] and segmentation [12–15], prognosis prediction [16, 17], and cancer diagnosis [18]. Recently, deep learning [19] has gained much attention due to its impressive performances in computer vision tasks. Deep learning methods are generally based on convolutional neural networks (CNNs) that learn features and prediction models with fewer human interventions than conventional machine learning and are also combined with fuzzy learning for robust feature representation [20] and reinforcement learning for self-taught decision-making [21].

As for automatic cancer cellularity assessment, there are two literatures addressing this challenge [22, 23]. In [22], Peikari et al. use a two-stage method consisting of nuclei segmentation and classification. The images extracted from whole slide images (WSIs) are preprocessed with decorrelation stretching for contrast enhancement, and the nuclei are segmented with multilevel Otsu's thresholding [24, 25], morphological operations, and marker-controlled watershed [26]. Then, two support vector machines (SVMs) trained using shape, textural, and spatial features extracted from the annotated nucleus figures are applied to distinguish the lymphocytes from epithelial nuclei and classify the epithelial as benign or malignant. The cellularity is estimated as the proportion of area filled with the cytoplasm of malignant epithelial cells. This procedure is analogous to the workflow of the pathologist; however, it is subject to the accuracy of segmentation and classification. In [23], Akbar et al. fine-tuned two modified Inception [27] networks independently to identify the images as cancerous or noncancerous and predict the cellularity for those cancerous patches. The method is validated using the same dataset as in [22], and superior performance is achieved. This method is based on end-to-end deep learning models, and the prediction of cellularity is directly given.

In recent literatures [28–30], transfer learning has also been applied to pathological images. Transfer learning is generally defined as the strategy to apply the knowledge obtained from one task to another related task [31, 32]. Practically, transfer learning makes training less dependent on the quantity of data utilizing the prior knowledge integrated in the models pretrained on a large scale dataset. In [29], the authors extract deep features from the top three layers of a pretrained AlexNet and use logistic regression to classify benign and malign tissue from breast cancer pathological slides. Weiss et al. [30] made a comparison on different features extracted from different models and found that the features from lower layers of Xception [33] perform the best. The basic idea of [28] is similar, but the authors used the lower layers of 3 different pretrained models for feature extraction and gradient boosting decision trees for classification. The performance improves compared to end-to-end CNN classifier.

In this work, we propose a novel framework to directly estimate cellularity from breast cancer histopathological slide patches combining deep feature representation, tree boosting, and SVM. Unlike the previous approach proposed by Peikari et al., our methods require for training only the cellularity labels on image patches instead of the annotations on individual nucleus. The following contributions are made:

1. Our method can directly estimate cellularity from breast cancer slide patches and avoid the segmentation and classification of nuclei, which is similar to the approach in [23].
2. We validate the transferability to tissue microscopy of the deep features learned from natural images. With the pretrained features, we manage to address the problem of data scarcity.
3. To tackle the problem of label imbalance, we make our prediction using regression and learn to rank model.

According to the experiments, we show that our methods are robust enough and state-of-the-art performances are achieved.

## 2. Materials and Methods

### 2.1. Materials

The data used in our research are acquired from the SPIE-AAPM-NCI BreastPathQ Challenge [34] and are from the same batch as those in [22, 23]. The dataset consists of 69 H&E stained WSI collected from the resection specimens of 37 post-NAT patients with invasive residual breast cancer. The specimens are processed following regular histopathological protocols, and the WSIs are scanned at 20x magnification (0.5 *μ*m/pixel). The tumor beds of these slides are roughly segmented into 4 regions (normal (0), low cellularity (1–30%), medium cellularity (31–70%), and high cellularity (71–100%)), and approximately equal numbers of patches are selected from each of them. In total, 2579 image patches with ROI of 512 × 512 pixels (about 1 mm^2^) are selected and labeled by an expert pathologist with manually estimated cellularity ranging between [0, 100%], as described in [22]. These patches are randomly partitioned by the challenge organizer into training set with 33 patients (2394 patches) and test set with 4 patients (185 patches). In our experiments, the test set is separated from training.

The bottom row of Figure 1 shows 4 examples from the test dataset with different cellularity and morphological features of benign epithelial, lymphocyte, and malignant epithelial nuclei. Besides, stromal nuclei are also presented in the images and are largely different in shape from the other nuclei. Nuclei presented in this dataset can be categorized as one of these 4 classes. Cellularity is defined as the percentage of the area of malignant epithelial cell.

**Figure 2:**
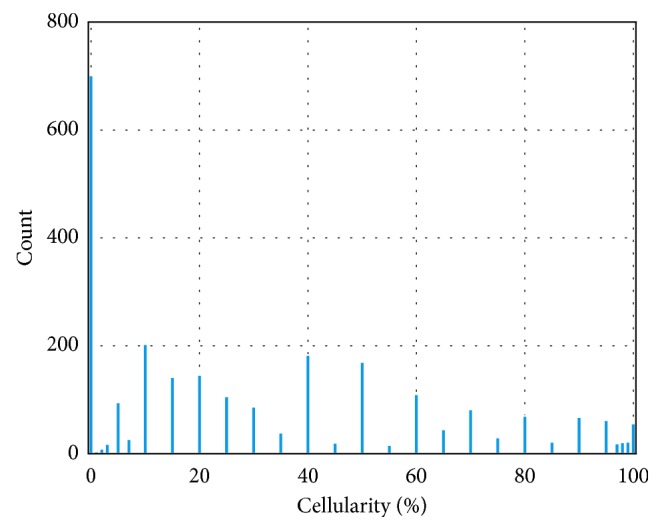
Histogram of cellularity distribution of the whole dataset.

The histogram of cellularity value on the whole dataset is presented in Figure 2, from which it is clear that there is heavy imbalance on cellularity distribution. The bin density varies in different intervals: the cellularity difference between neighboring bins is 5% in [10%, 90%], whereas in [0, 10%) ∪ (90%, 100%], it is 1%. Numbers of patches varies among the bins: 705 patches are labeled as 0 cellularity; 14 of the bins contain 50–200 patches; 22 of them contain fewer than 50 patches; some of the bins contain no patches (4%, 6%, 9%, 91%, 94%, 96%).

### 2.2. Methods

#### 2.2.1. Overview

There are two major problems in this project. The first problem is data scarcity, as only less than 3000 samples from 37 patients are included in our dataset. This could be addressed using transfer learning [35]. Transfer learning reduces the dependency on data quantity with the prior knowledge learned from another dataset that has proved transferability and generalizability to other scenarios [31, 32]. Generally, there are two options to apply transfer learning in medical image: (1) to fine-tune the pretrained CNN; (2) to use the pretrained CNN as feature extractor and combine it with conventional machine learning [36]. The second problem, label imbalance, as shown in Figure 2 where cellularity is not uniformly distributed in all the discrete bins, poses a challenge to learning algorithms susceptible to data distribution. In this work, we solve this problem using regression and learn to rank models.

**Figure 3:**
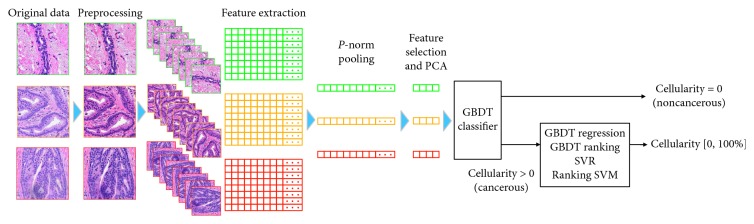
Overview workflow of our methods. The images are first preprocessed to obtain consistent stain. Thousands of features are extracted from each image using the pretrained CNNs and are pooled to enhance rotation invariance. A few hundred features are obtained from mRMR feature selection and PCA and are used to train GBDT and SVM.

We propose a framework that holds promise to overcome the two problems stated above following the second option of transfer learning. As illustrated in Figure 3, the workflow of our methods consists of the following steps: (1) data preprocessing that includes stain normalization and data augmentation, as detailed in Section 2.2.2; (2) deep feature representation that extracts thousands of features from each patch, as detailed in Section 2.2.3; (3) feature selection via minimum redundancy maximum relevance (mRMR) [37] and dimensionality reduction by principal component analysis (PCA), as detailed in Section 2.2.4; (4) training gradient boosting decision trees (GBDT) classifier using the refined features to distinguish between cancerous and noncancerous patches, as detailed in Section 2.2.5; (5) training GBDT and SVM to predict the cellularity for those cancerous patches, as detailed in Sections 2.2.6 and 2.2.7. The metrics to evaluate the performance of our methods are briefly described in Section 2.2.8.

#### 2.2.2. Data Preprocessing

Stain inconsistency of digital WSI due to fixation, embedding, cutting, and staining of tissue sections is common among slides from different microscopes and different staining batches [38]. This contributes to very negative effect on quantitative analysis. To reduce stain variation in our dataset, we normalize the H&E stain on the slide patches with the algorithm proposed in [39]. It first converts the RGB image to optical density (OD) space and finds the optimal stain vectors for H and E by calculating singular value decomposition (SVD) on the OD tuple, a *N* × 3 matrix, where *N* is the number of pixels and 3 represents the RGB channels. Then, the image is deconvolved using these stain vectors and normalized using 2 predefined reference vectors.

Generally, data augmentation is indispensable to reduce overfitting in statistical learning systems. Following the method proposed in [28, 40], we first convert the color of the tissue from RGB space to H&E space, then we multiply each channel by a factor randomly and uniformly sampled from range [0.7, 1.3]. Other commonly used augmentations, such as cropping and rescale, are ineligible for this task as rescale causes loss in resolution and cropping varies the exact cellularity when cancerous or noncancerous regions are cropped out.

#### 2.2.3. Deep Feature Representation

**Figure 4:**
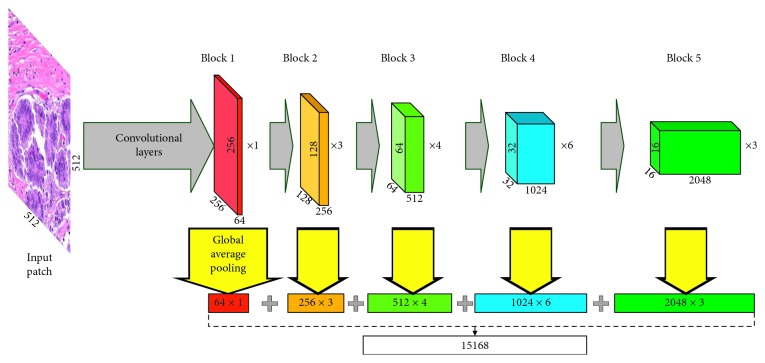
Schematic diagram of feature extraction with ResNet.

In our method, deep features are extracted from the augmented patches with CNN pretrained on ImageNet [41], which is similar to [28]. Three architectures, namely, VGG-16 [42], ResNet-50 [43], and Inception-v3 [27], are applied. As suggested in [44], lower layers of deep learning models are expected to preserve more generic and transferable features. Therefore, we focus on the convolutional layers of these models. Global average pooling is used in each layer to decrease the dimensionality of features. In Figure 4, ResNet is taken as example to show our scheme of feature extraction. In total, 4224, 15168, and 10048 features are to be extracted from VGG, ResNet, and Inception, respectively. With rotation (0, 90°, 180°, and 270°) and flipping, 8 variations are to be generated from each patch, so that 8 different feature vectors are to be extracted. To obtain rotation invariance, we combine them into one vector by applying the *p*-norm pooling approach on each dimension [45, 46], which can be formulated as

(1) $$\begin{array}{c} f_{\text{pooling}}={\left(\frac{1}{N}\overset{N}{\underset{i=1}{\sum }}{\left(f_{i}\right)}^{p}\right)}^{1/p}, \end{array}$$

where *f* is the feature dimension to be pooled, *N*=8 is the number of vectors, and *p* is the norm, which is set to 3.

#### 2.2.4. Feature Selection and Dimensionality Reduction

We apply the mRMR feature selection method suggested in [37] to search for the fewest features that preserve sufficient information for prediction. The basic idea of mRMR is to select a feature subset where the features are marginally as correlated to the target variable as possible while mutually as uncorrelated to each other as possible. Practically, mRMR is developed to be a filter that iteratively selects features with greedy algorithm based on the mutual information between the target variable and each feature. In this work, mutual information quotient (MIQ) is chosen as the feature filtering criterion. The performances of using different numbers of top ranking features (*n*_feat_) are evaluated to determine the optimal *n*_feat_.

PCA is used to reduce the dimensionality of the selected features. PCA transforms the features into uncorrelated components, among which those with the smallest variance are considered noisy and are thus abandoned. In general, feature selection and dimensionality reduction eliminate irrelevant information and speedup training.

#### 2.2.5. Tissue Classification Based on GBDT

Boosting is an ensemble method where new models are added serially to minimize the loss of previous models until no further improvements can be made. In this procedure, a series of weak models are combined to make up a stronger one with the primary goal to reduce bias and variance. GBDT [47] is a boosting approach where new decision trees are trained to fit the residuals of the existing trees and then added together to optimize the object function. It is a powerful and popular technique that has been used in many leading solutions to data science challenges with success in classification and regression. The name gradient boosting comes from the gradient descent algorithm that is used to minimize the loss when adding new models. XGBoost [48] is an implementation of GBDT developed for faster speed and superior performance. Remarkable features of XGBoost include parallelization of tree construction, distributed computing, out-of-core computing, and cache-aware prefetch. LightGBM [49] is another gradient boosting framework achieving higher efficiency and lower memory usage with the use of histogram-based splitting, leafwise tree growth, and optimization of parallel. Both XGBoost and LightGBM support L1, L2, and model complexity regularization in order to reduce overfitting.

As illustrated in Figure 2, our data are heavily imbalanced. As the patches with zero cellularity, i.e., noncancerous tissue, make up nearly 1/4 of the training samples, sifting them out helps balancing the dataset and improving regression performance. In this section, we describe our GBDT-based method to classify the patches as cancerous or noncancerous. In general, GBDT aims to minimize a predefined loss function. For the target of binary classification, binary cross entropy (BCE) is optimized, which can be formulated as

(2) $$\begin{array}{c} L_{\text{BCE}}=-\overset{M}{\underset{i=1}{\sum }}y_{i}\;\log\left(\sigma \left(F\left(x_{i}\right)\right)\right)+\left(1-y_{i}\right)\log\left(1-\sigma \left(F\left(x_{i}\right)\right)\right), \end{array}$$

where *F*(**x**_**i**_) is the output of the trees for sample **x**_**i**_, *M* is the number of training samples, *σ* is the sigmoid function, and *y*_*i*_ ∈ {0,1} is the training label. BCE is a smooth and differentiable function, which is a prerequisite to be optimizable for GBDT. Our model is trained using the selected and reduced features.

#### 2.2.6. Prediction for Cancerous Patches Using GBDT

As described in Section 2.2.5, GBDT is a powerful model that hold promises to solve both classification and regression problems. To predict the cellularity on a continuous scale for those cancerous patches using the selected features, 2 kinds of loss are optimized.

*(1) Huber Loss*. Huber loss can be formulated as

(3) $$\begin{array}{c} L_{\text{Huber}}=\overset{M}{\underset{i=1}{\sum }}h_{i}, \end{array}$$

where

(4) $$\begin{array}{c} h_{i}=\left\{\begin{array}{cc} \frac{1}{2}{\left(y_{i}-F\left(x_{i}\right)\right)}^{2}, & \left|y_{i}-F\left(x_{i}\right)\right|\leq \delta ,\\ \delta \left|y_{i}-F\left(x_{i}\right)\right|-\frac{1}{2}\delta^{2}, & \text{otherwise}, \end{array}\right. \end{array}$$

where *y*_*i*_ is the target variable, *F*(**x**_**i**_) is the model prediction on sample **x**_**i**_, *M* is the number of training samples, and *δ* is the parameter controlling the steepness of Huber function.

It is clear that Huber loss is actually a combination of L1- and L2-norm loss. In our regression models, the target variable *y*_*i*_ is cellularity.

*(2) Ranking Loss*. In information retrieval, RankNet [50] is a supportive machine learning model that solves the ranking problems. Generally speaking, a RankNet is intended to output a ranking score indicating the ranking position given an input feature vector.

Analogous to the manual workflow, the cellularity of an object image patch could be estimated by comparing it to the patches of known cellularity. This problem could be probably handled using the pairwise ranking method. In this task, we apply the pairwise RankNet [50], which trains a ranking model by optimizing the loss function based on pairs of feature vectors in adjacent levels. Prior to training, different cellularities are first translated into different levels. The cellularity 0, 1%, 2%,…, 10% are translated to level 0, 1, 2,…, 10, respectively. 15%, 20%, 25%,…, 90% are translated to 11, 12, 13,…, 26 and 91%, 92%, 93%,…, 100% to 27, 28, 29,…, 36, respectively. The probability that feature vector **x**_**i**_ should be ranked higher than **x**_**j**_ (i.e., the cellularity of **x**_**i**_ is lower than that of **x**_**j**_) can be formulated as

(5) $$\begin{array}{c} P_{ij}=\sigma \left(k\left(F\left(x_{i}\right)-F\left(x_{j}\right)\right)\right), \end{array}$$

where *σ* is the sigmoid function, *F* is the ranking model, and *k* controls the shape of sigmoid. The ranking loss is defined as the sum of BCE of *P*_*ij*_:

(6) $$\begin{array}{c} L_{\text{rank}}=\underset{i,j\in A}{\sum }-{\bar{P}}_{ij}\;\log P_{ij}-\left(1-{\bar{P}}_{ij}\right)\log\left(1-P_{ij}\right), \end{array}$$

where ${\bar{P}}_{ij}\in \left\{0,1\right\}$ denotes the true ranking between **x**_**i**_ and **x**_**j**_ and *A* denotes the set of any feature vector pairs in adjacent cellularity levels.

**Figure 5:**
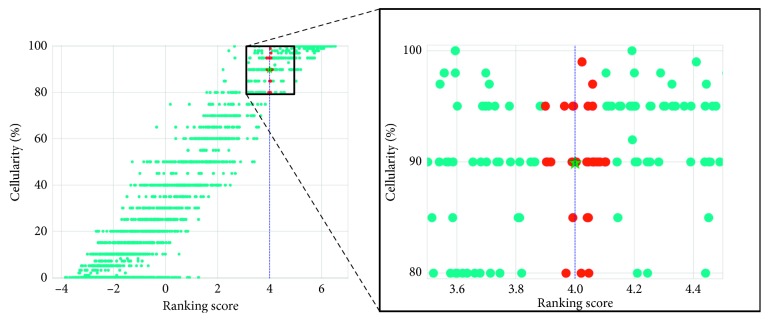
Illustration of our KNN method to map the ranking scores to cellularity. The red dots represent the training samples that lie within the 30-nearest neighborhood of a testing samples with ranking score 4.0. The cyan dots represent those outside the neighborhood. The prediction for the testing sample is the mean of cellularity of all the red dotted samples, as denoted by the green star.

The output ranking scores are recalibrated to [0, 100%] using K-nearest neighborhood (KNN) (*k* = 30), where the ranking scores are mapped to cellularity with reference to the scores of the training set. The illustration of our KNN mapping method is presented in Figure 5.

#### 2.2.7. Prediction for Cancerous Patches Using SVM

SVM is a classical model that provides promising solutions to classification and regression problems. Similar to GBDT, we propose methods based on support vector regression (SVR) [51, 52] and ranking SVM [53] for the estimation of cellularity for those cancerous patches.

*(1) SVR*. SVR is a linear prediction model that tries to minimize the largest margin produced by the training samples by penalizing the outliers using *ε*-insensitive loss. Those samples that lie beyond the *ε* margin are called support vectors. Similar to SVM classifier, the training of SVR can be formulated as

(7) $$\begin{array}{c} \begin{array}{cc} \underset{w,b,\xi_{i},{\hat{\xi }}_{i}}{\min} & \frac{1}{2}{\left\|w\right\|}^{2}+C\overset{M}{\underset{i=1}{\sum }}\left(\xi_{i}+{\hat{\xi }}_{i}\right),\\ \text{s}.\text{t}. & f\left(x_{i}\right)-y_{i}\leq \varepsilon +\xi_{i},\\ & y_{i}-f\left(x_{i}\right)\leq \varepsilon +{\hat{\xi }}_{i},\\ & \xi_{i}\geq 0,{\hat{\xi }}_{i}\geq 0,\;i=1,2,\ldots ,M, \end{array} \end{array}$$

where *f*(**x**_**i**_)=**w** ^**T**^ **x**_**i**_+*b* is the linear regression model, *y*_*i*_ is the target variable, *ξ*_*i*_ and ${\hat{\xi }}_{i}$ are the slack variables, *C* is the tradeoff parameter, and *ε* controls the width of the margin. This problem can be solved using the classical Lagrange duality and KKT conditions. Kernel tricks can be implemented by replacing **x**_**i**_ with Φ(**x**_**i**_), and the most commonly used kernels are linear kernels and radial basis functions (RBF). By setting *ε* as 0 and substituting *ξ*_*i*_^2^ and ${\hat{\xi }}_{i}^{2}$ for *ξ*_*i*_ and ${\hat{\xi }}_{i}$ in the loss function, SVR is equivalent to L2-regularized linear regression. In our method, the target variable is cellularity.

**Figure 6:**
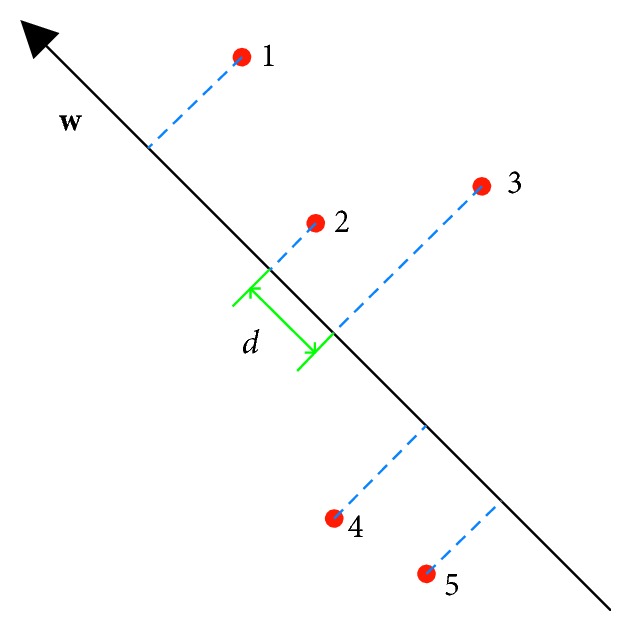
Illustration of Ranking SVM using 2D features, where **w** is the weight vector, 1–5 are the training samples, and *d* is the minimal margin that should be maximized.

*(2) Ranking SVM*. As stated in Section 2.2.6, the problem of cellularity estimation could be handled with ranking models. We use ranking SVM [53] for this problem. Ranking SVM tries to build a linear ranking model using weight vector **w** and minimizes the pairwise ranking error by maximizing the minimal margin between pairs of ranking scores, as illustrated in Figure 6, where the minimum distance *d* on the weight vector **w** between any two samples is maximized. Based on the idea of SVM classifier, ranking SVM can be formulated as a convex optimization problem:

(8) $$\begin{array}{c} \begin{array}{cc} \underset{w,\xi_{i,j}}{\min} & \frac{1}{2}{\left\|w\right\|}^{2}+C\sum \xi_{i,j},\\ \text{s}.\text{t}. & w^{\text{T}}x_{i}-w^{\text{T}}x_{j}\geq 1-\xi_{i,j},\\ & \xi_{i,j}\geq 0,\forall \left(x_{i}⊳x_{j}\right), \end{array} \end{array}$$

where **w** ^T^ **x**_**i**_ and **w** ^T^ **x**_**j**_ are the ranking scores for **x**_**i**_ and **x**_**j**_, *C* is the tradeoff parameter, *ξ*_*i*,*j*_ is the slack variable, and **x**_**i**_ ⊳ **x**_**j**_ denotes that **x**_**i**_ should be ranked higher that **x**_**j**_. Prior to training, we translate cellularity into different ranking levels, the same as in Section 2.2.6. The ranking scores predicted by SVM are mapped to cellularity using the KNN method described in Section 2.2.6.

#### 2.2.8. Evaluation Metrics

To evaluate the performance of different methods on the test set using the manual labels as reference, 3 metrics are used: intraclass correlation (ICC), Kendall's tau-b (*τ*_*b*_) [54, 55], and the prediction probability (*P*_*K*_) [56].

The ICC in this work refers to ICC (A, 1) according to the definitions in [57, 58]. It uses two-way model of analysis of variance (ANOVA) to evaluate the absolute agreement between any two measurements and can be formulated as

(9) $$\begin{array}{c} \text{ICC}=\frac{{\text{MS}}_{R}-{\text{MS}}_{E}}{{\text{MS}}_{R}+\left(k-1\right){\text{MS}}_{E}+k/n\left({\text{MS}}_{C}-{\text{MS}}_{E}\right)}, \end{array}$$

where MS_*R*_ denotes the mean square for rows (testing samples), MS_*E*_ denotes the mean square error, MS_*C*_ denotes the mean square for columns (measurements), *k*=2 is the number of measurements, and *n*=185 is the number of testing samples.

Kendall rank correlation coefficient is a metric used to evaluate the ordinal correlation between two measurements with nonparametric hypothesis test [55]. It is calculated based on the measurements of pairs of testing samples. In this work, we use Kendall's tau-b [54], which is adjusted for ties and can be formulated as

(10) $$\begin{array}{c} \tau_{b}=\frac{n_{c}-n_{d}}{\sqrt{\left(n-n_{T_{x}}-n_{T_{xy}}\right)\left(n-n_{T_{y}}-n_{T_{xy}}\right)}}, \end{array}$$

where *x* denotes the automatically estimated cellularity, *y* denotes the manual measurement, *n*_*c*_ and *n*_*d*_ denote the number of concordance and discordance pairs, respectively, *n*_*T*_*x*__ denotes the number of ties only in *x*, *n*_*T*_*y*__ denotes the number of ties only in *y*, and *n*_*T*_*xy*__ denotes the number of ties in both *x* and *y*.

Prediction probability [56] is the metric adopted by the challenge organizer. It also evaluates the ordinal correlation and can be formulated as

(11) $$\begin{array}{c} P_{K}=\frac{n_{c}+n_{T_{x}}/2}{n_{c}+n_{d}+n_{T_{x}}}. \end{array}$$

As explained by the challenge organizer, the reason for evaluating automatic methods with ordinal correlation metrics is that there exists variability among expert pathologists on cellularity assessment, and thus it is hard to define a calibrated and unbiased cellularity reference. Moreover, the cellularity estimated by the algorithm can be normalized to [0, 100%] without loss in consistency.

## 3. Experiments and Results

The feature extraction is carried out using Keras with TensorFlow backend, with the support of a NVIDIA GTX 1080 Ti GPU. All parameters of the pretrained models are provided by their respective authors.

### 3.1. Feature Selection

**Figure 7:**
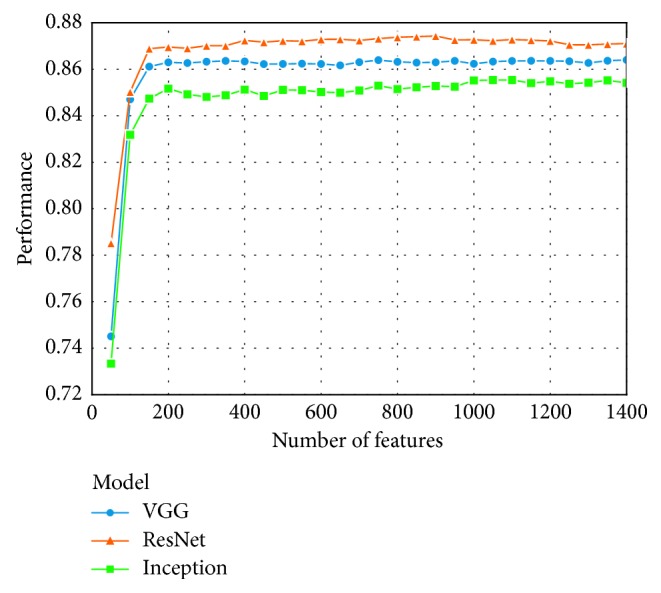
The curves showing the relation between model performances and the number of features.

We perform feature selection using MIQ as criterion and sort the features into a list according to their prediction potential. Then, different numbers of top ranking features are selected to train GBDT models with Huber loss, and the performance is evaluated using *P*_*K*_ with 9-fold cross-validation on the training set. The parameter setting for GBDT is listed in Table 1. The relation between model performances and the number of features is illustrated in Figure 7. If not specified, the number of features selected from VGG, ResNet and Inception are 400, 800, and 1000, respectively, in the following experiments.

### 3.2. Tissue Classification Based on GBDT

**Figure 8:**
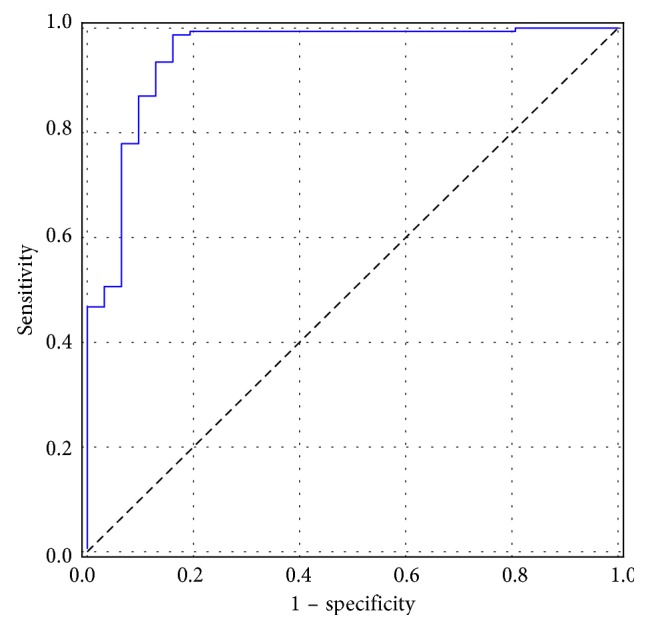
The ROC curve of our GBDT classification.

Our GBDT classifier is constructed with the LightGBM package and is trained using the ResNet features that are not processed with mRMR and are processed with PCA where 40 principal components are preserved. Three hundred decision trees with depth of 7 are constructed, with the primary goal to minimize the BCE loss. The feature fraction and bagging fraction are 0.6 and 0.8, respectively, and the learning rate is 0.01. The other parameters are set as default. The ROC curve of our classification on the test set is presented in Figure 8, and the area under curve (AUC) is 0.95. This classifier can detect cancerous images in the test set with sensitivity of 0.98, specificity of 0.84, and accuracy of 0.96.

### 3.3. Prediction for Cancerous Patches

For tree boosting regression using Huber loss, we construct GBDT with the LightGBM package using the augmented training set. The *δ* in Huber loss is set to 1. For RankNet, we train the model with the XGBoost package. The parameter settings are summarized in Table 1, and parameters not listed are set as default. The features used to train the GBDTs are selected using mRMR and are not processed by PCA.

For SVR, we use LIBSVM [59] wrapped in the Scikit-learn [60] package. The top 4000, 8000, and 4000 features are selected from VGG, ResNet, and Inception, respectively, and the number of components for PCA is set as 150. The tradeoff parameter *C* is set as 200, and RBF with *γ*=0.0001 is used as the kernel for the SVR with *ε*=0.

For Ranking SVM, we use the software provided in [53]. The top 50 features are selected from each CNN, and the number of components for PCA is set as 20. Linear kernel is used, and the tradeoff parameter *C* is set as 1*e*8.

Linear correction is used to make the mean and standard deviation of the estimated cellularity equal to those of the manual labels of the training set. All the parameter settings in our experiments are tuned using 9-fold cross-validations on the training set.

The classifier is first used to identify cancerous patches, and regression models combined with different features are applied to further predict the cellularity for them. Complete evaluations of our methods on the test set are listed in Table 2, from which it is clear that SVR combined with ResNet features obtains the best performance. With high correlation scores and tight upper and lower bounds of the 95% confidence interval, this method shows good agreement with the pathologist and produces stable predictions. Overall, the features from ResNet are more robust than the others, and SVR is the best model.

The ICC of the methods proposed by Peikari et al. [22] and Akbar et al. [23] are reported as 0.74 [0.70, 0.77] and 0.83 [0.79, 0.86], significantly lower than those of many of our models. The ICC between some of our methods and the pathologist's estimation is even higher than that between two different pathologists (0.89 [0.70, 0.95]) reported in [22], indicating that our methods can possibly supplant manual estimation in clinical practice.

**Figure 9:**
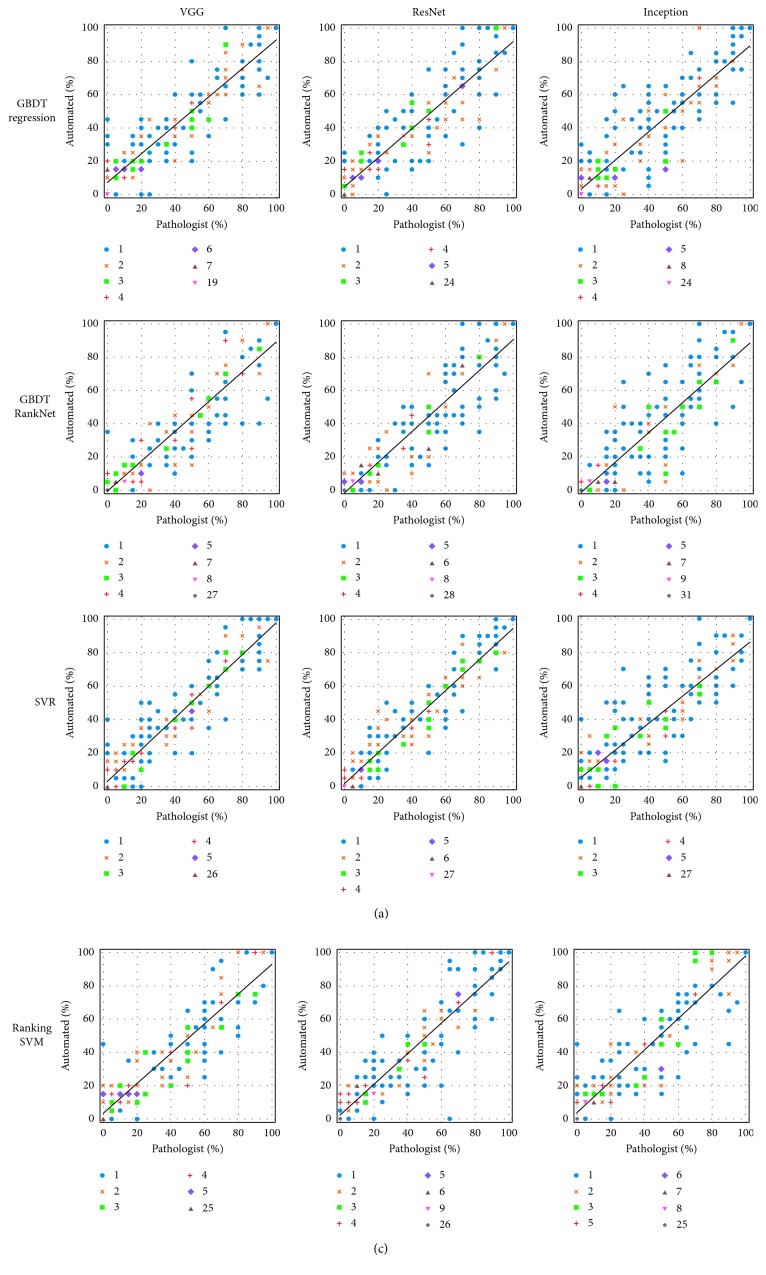
Scatter plots showing the agreement between the human pathologist and the automated approaches. The marker style of each dot indicates the number of patches with the corresponding manually and automatically estimated cellularity.

The scatter plots in Figure 9 show the agreement on cellularity estimation between the pathologist and our automated methods. Note that to make the plots clear, each estimated cellularity is rounded to its nearest discrete bin. As presented in the figure, our predictions are in good agreement with the assessment by the pathologist. Generally, most of the estimations made by our methods are close to those by the pathologist. The black lines show the linear regressions between manual and automated measurements. As can be seen, the slopes of the regression lines of SVR and ranking SVM methods are close to 1, indicating good correlations to manual estimation.

### 3.4. Comparisons to the Method by Peikari et al

For direct comparisons to the method proposed by Peikari et al. [22] using exactly the same test set, we implement their algorithm. We follow their instructions for nuclei segmentation, labeling and classification, and cellularity computation. The dataset of manually annotated nuclei is provided by them as a part of the challenge. We compute 2 thresholds for each image using the Otsu algorithm and the lower one is used for segmentation. Morphological opening with a disk with radius of 3 is used to smooth nuclei boundaries and separate neighboring objects. Distance transform and local maxima finding are used to locate the centroids of overlapping nuclei, and marker-controlled watershed is applied to separate them. According to our observation and the proportion of matched nuclei, this parameter setting obtains optimal segmentation performance. Stromal nuclei are eliminated from classification by removing objects with ratio of major to minor axes ≥3. In total, 21573 nuclei (close to the 21799 nuclei in [22]) are matched to manual annotations and are partitioned into training set (13945 nuclei) and test set (7628 nuclei). The test set is separated from training and preserved to evaluate the generalization performance of the classifiers. Based on 5-fold cross-validation on the training set, we set *C*=100 and *γ*=0.01 for the two SVMs.

**Figure 10:**
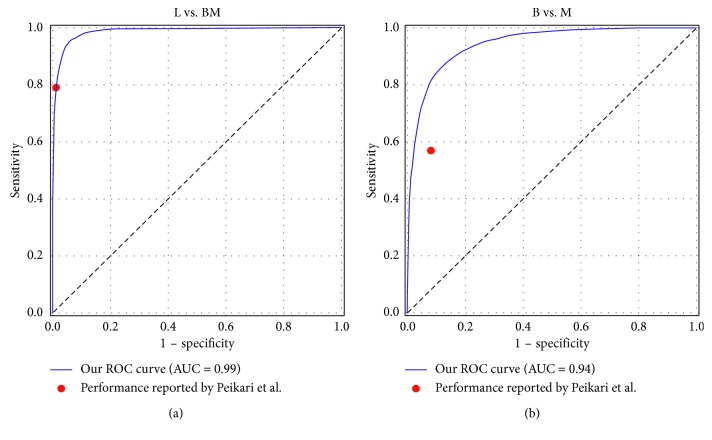
The ROC curves of our nuclei classification based on 5-fold cross-validation on the training set.

Our performance of 5-fold cross-validation on the training set of nuclei classification is presented in Figure 10, where the ROC curves of distinguishing lymphocyte from epithelial nuclei (L vs. BM) and classifying benign and malignant epithelial nuclei (B vs. M) are shown. It is clear that the points representing the performances reported by Peikari et al. locate on or under our ROC curves and that our AUCs are 0.99 and 0.94, trivially higher than those reported by them (0.97 and 0.86). The performance of our classifiers on the test set is summarized in Table 3. Comparing it to Table 3 of [22], it can be concluded that our implementation achieves comparable classification performance. We apply the nuclei detection and the postprocessing methods (including KNN correction and morphological dilation) described by Peikari et al. on the 185 testing samples for cellularity estimation and the ICC, *τ*_*b*_ and *P*_*K*_ are 0.76 [0.69, 0.81], 0.57 [0.49, 0.63], and 0.79 [0.75, 0.82], respectively, consistent with the performance reported in [22] and significantly outperformed by our methods.

## 4. Discussion

Cancer cellularity is an important component in the assessment of RCB, an effective indicator of tumor response. In this work, we propose a novel framework based on pretrained CNN features, GBDT, and SVM for direct cellularity estimation from histopathological slide patches within the tumor bed of breast cancer. Prior to training, feature selection is implemented to eliminate irrelevant features and PCA is used to reduce dimensionality, in order to reduce overfitting and improve training efficiency. Our methods are validated on a dataset consisting of 185 image patches and obtain state-of-the-art performance in terms of agreement with the human pathologist, in comparison to two other methods [22, 23]. The agreements between our methods and the pathologists' are even better than that between different pathologists, thus they are potential substitutes for manual inspection in clinical practice.

The problem of data scarcity is overcome using transfer learning. We extract deep features from multiple layers and manage to construct GBDT and SVM with a limited quantity of data. The results of our method demonstrate the transferability of deep features learned from natural images to specific histopathological data. The validity of general deep features on pathological images is also verified by recent literatures [28–30], where other tasks are handled.

The dataset poses a great challenge of label imbalance: the cellularity is not uniformly distributed in all the discrete bins. Two strategies are used in our framework to solve this problem. First, the noncancerous patches are sifted out using a GBDT classifier, as these patches make up a large part of our data. Second, regression and learn to rank models are used to predict the cellularity for the cancerous patches, as these models are insusceptible to such imbalance.

The approach proposed by Peikari et al. [22] is a two-stage method including segmentation and classification of individual nucleus with hand-crafted features, thus the performance depends heavily on the accuracy of nuclei identification and additional datasets of annotated nuclei are necessary for training. These drawbacks are overcome by the methods proposed by us and Akbar et al., where direct predictions for cellularity are given and only manual labels of cellularity are necessary. In [23], Akbar et al. fine-tuned two modified CNN for classification and regression. It can be concluded that direct learning models are generally more robust than nuclei detection-based estimations. There are two major differences between our methods and that reported by Akbar et al. First, the features extracted by our deep models are processed with more advanced learning algorithms, while in their method, predictions are based on linear models. Second, our methods extract features from multiple levels of CNN while their predictions are purely based on the last layer. The results show that our methods achieve better agreement with the human pathologist.

It is reasonable to suppose that our methods are ready to be adapted to other histological applications as long as the corresponding data and cellularity labels are provided. The cellularity distribution over breast cancer tumor bed can be easily mapped, and the post-NAT tumor burden can be assessed in a semiautomatic workflow. Future research should focus on the automated segmentation of tumor bed and the estimation of other parameters of RCB.

## 5. Conclusion

In this work, novel methods for direct cellularity estimation combining deep feature representation, tree boosting, and SVM are proposed. The agreements between the estimations by our methods and those by human pathologists are validated using 3 metrics. Furthermore, the training of our models requires only lightly labeled data instead of annotations on individual nuclei, thus is more generalizable.

## Figures and Tables

**Table 1:** Parameter settings of the GBDT for cancerous patches.

Parameter	Huber	RankNet
Maximum tree depth	4	3
L1 regularization	0.001	0.001
L2 regularization	0	0
Feature fraction	0.6	0.6
Bagging fraction	0.7	0.8
Learning rate	0.01	0.01
Number of trees	1500	1000

**Table 2:** Evaluations of all our methods.

Model	Feature	ICC (95% CI)	*τ*_*b*_ (95% CI)	*P*_*K*_ (95% CI)
GBDT with Huber loss	VGG	0.91 [0.87, 0.93]	0.78 [0.69, 0.79]	0.90 [0.86, 0.91]
	ResNet	0.91 [0.87, 0.93]	0.77 [0.73, 0.81]	0.90 [0.87, 0.91]
	Inception	0.87 [0.84, 0.90]	0.71 [0.65, 0.76]	0.86 [0.84, 0.89]

GBDT with RankNet	VGG	0.91 [0.88, 0.93]	0.77 [0.72, 0.81]	0.89 [0.87, 0.91]
	ResNet	0.91 [0.88, 0.94]	0.79 [0.75, 0.82]	0.90 [0.88, 0.92]
	Inception	0.88 [0.85, 0.91]	0.74 [0.70, 0.79]	0.88 [0.86, 0.90]

SVR	VGG	0.94 [0.92, 0.95]	0.80 [0.76, 0.84]	0.91 [0.89, 0.93]
	ResNet	**0.95 [0.93, 0.96]**	**0.83 [0.79, 0.86]**	**0.93 [0.91, 0.94]**
	Inception	0.89 [0.85, 0.92]	0.74, [0.69, 0.79]	0.88 [0.85, 0.90]

Ranking SVM	VGG	0.91 [0.88, 0.93]	0.76 [0.71, 0.80]	0.89 [0.87, 0.91]
	ResNet	0.92 [0.89, 0.94]	0.78 [0.73, 0.82]	0.90 [0.88, 0.92]
	Inception	0.89 [0.86, 0.92]	0.76 [0.71, 0.80]	0.89 [0.86, 0.91]

Bold indicates the best performance in terms of the corresponding metric.

**Table 3:** Performance of the SVMs on the independent test set for nuclei classification.

Class	Accuracy (%)	Sensitivity (%)	Specificity (%)
Lymphocyte (L)	95	84	98
Benign epithelial (B)	88	60	93
Malignant epithelial (M)	89	93	82

## Data Availability

The histopathology image data used to support the findings of this study have been deposited in http://spiechallenges.cloudapp.net/competitions/14.
